# Acid Hydrolysis of Pectin and Mucilage from Cactus (*Opuntia ficus*) for Identification and Quantification of Monosaccharides

**DOI:** 10.3390/molecules27185830

**Published:** 2022-09-08

**Authors:** Vanessa Garfias Silva, María Soledad Cordova Aguilar, Gabriel Ascanio, Juan Pablo Aguayo, Karen Y. Pérez-Salas, Ana del Carmen Susunaga Notario

**Affiliations:** 1 Facultad de Química, Universidad Nacional Autónoma de México, Circuito Exterior S/N, Ciudad Universitaria, Coyoacan, Mexico City 04510, Mexico; 2 Instituto de Ciencias Aplicadas y Tecnología, Universidad Nacional Autónoma de México, Circuito Exterior S/N, Ciudad Universitaria, Coyoacan, Mexico City 04510, Mexico; 3 Programa de Maestría y Doctorado en Ingeniería, Universidad Nacional Autónoma de México, Circuito Exterior, Ciudad Universitaria, Coyoacan, Mexico City 04510, Mexico; 4 Cátedra CONACyT-Instituto de Ciencias Aplicadas y Tecnología, Universidad Nacional Autónoma de México, Circuito Exterior S/N, Ciudad Universitaria, Coyoacan, Mexico City 04510, Mexico

**Keywords:** pectin, mucilage, hydrocolloid, polysaccharide, cactus, hydrolysis, chromatography, phenol–sulfuric acid method

## Abstract

Pectin and mucilage are polysaccharides from the cactus *Opuntia ficus-indica*, which are also known as hydrocolloids, with useful properties in industries such as food, pharmaceuticals, and construction, among others. In the present work, cactus hydrocolloids were hydrolyzed characterized using two techniques: first, thin-layer chromatography, to identify the monosaccharides present in the sample, followed by the phenol–sulfuric acid method to determine the monosaccharide content. The hydrolyzing method allowed us to reduce the processing time to 180 min and, considering the identification and quantification procedures, the proposed methodology is much simpler and more cost-effective compared to other methods, such as high-performance liquid chromatography (HPLC), nuclear magnetic resonance (NMR), and mass spectrometry. The analysis of the results revealed that the maximum concentration of monosaccharides was obtained after hydrolyzing for 90 min. Under such conditions, with pectin being the main component contained in the cactus hydrocolloids analyzed here, galacturonic acid was found in the largest quantities.

## 1. Introduction

The cactus *Opuntia ficus-indica* (L.) *Miller*, commonly known as the prickly pear or cladode, is a vegetable that is widely consumed in Mexico. It is part of the cacti family that covers around 30% of the continental surface of the semi-arid and arid world [1].There are several factors that allow the cladodes to grow almost everywhere throughout the year and remain perpetually green despite the harsh environmental conditions; among these factors are the metabolism of the species and its peculiar adaptations to water scarcity and solar radiation, the acid metabolism of the Crassulaceae, the reduction in foliar tissues, and the cuticular waxes that cover the cladodes and the surface of the fruits. Due to their nutritional properties, cladodes have a variety of uses, giving them an added economic value. In addition, cacti are plants with diverse uses, such as food, ornamentation, foraging, construction, cosmetics, and medicine, among others [2,3,4,5,6,7,8,9,10].

The main constituent of *O. ficus-indica* cladodes is water (80–95%), followed by carbohydrates present as monosaccharides (3–7%), fiber (1–2%), and protein (0.5–1%). Cactus cladodes contain a mix of two polysaccharides: mucilage and pectin, which are also known as hydrocolloids. Such hydrocolloids can be used as materials for the development of edible biopolymers, since these can help improve the quality and increase the shelf life of different fruit and vegetable products [3,9,10,11,12,13,14,15,16].

**Figure 1:**
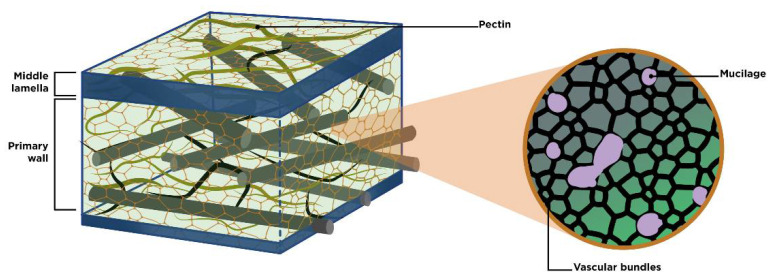
Location of pectin and mucilage in the cell wall.

**Figure 2:**
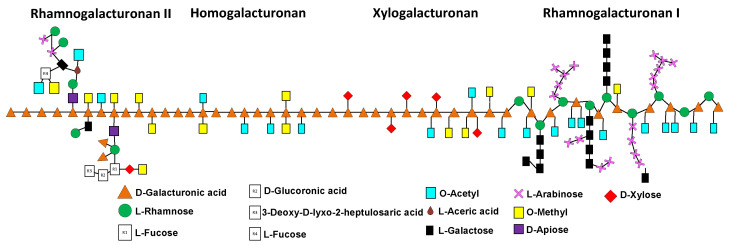
Pectin’s structure. Adapted from Harholt, J., Suttangkakul, A. and Vibe Scheller, H (2010) [23].

Pectin belongs to the group of heteropolysaccharides, and is present in all plants’ primary cells. it is derived from the breakdown of more complex protopectins, which can be found in the middle lamella of the cell wall (Figure 1). It functions as a moisturizing agent and cementing material for the cellulose networks. The backbone of pectin is mainly made up of D-galacturonic acid and, to a lesser extent, rhamnose. This linear chain has branches to other monosaccharides such as arabinose and galactose linked to the occupied sites of rhamnose in the main chain. On the other hand, xylose is directly linked to galacturonic acid. Figure 2 shows the structure of pectin in detail [15,16,17,18,19,20,21,22].

**Figure 3:**
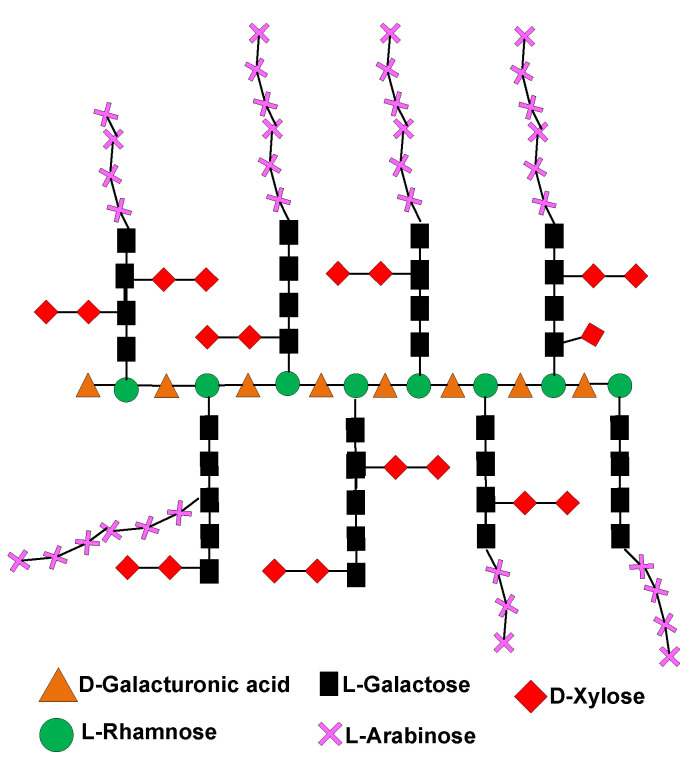
Mucilage’s structure. Adapted from McGarvie, D. and Parolis, H. (1981) [5].

Mucilage is a vegetal product containing L-arabinose, D-galactose (pyranose and furanose forms), D-xylose, L-rhamnose, glucuronic acid and, as the principal neutral sugar unit, D-galacturonic acid (Figure 3) [4,8,9,24,25,26,27]. It is located in extracellular spaces (Figure 1), synthesized from the polymerization of several monosaccharides associated with uronic acids [7,14], and excreted into the apoplast, forming a donut-shaped pocket between the membrane and the cell wall [9], where it helps to regulate cellular water content during the initial phase of dehydration [14,15,18].

The chemical structure (see Figure 3) shows great similarities to the highly branched regions of cell-wall pectin, for this reason mucilage from *O. ficus-indica*, are indistinctly referred to as pectin [13,14]. However, pectin is richer in galacturonic acid than mucilage [6,13,17,18,20,21,25].

The main components of both pectin and mucilage are the monosaccharides, which are involved in biological processes and functional applications [8,10,28,29,30,31,32,33,34,35,36,37,38,39]. These monosaccharides have several functions; for example, galactose is a physiological constituent of chemical compounds such as cerebrosides and gangliosides, which are essential in the nervous tissues of the brain. Mannose is used as a food supplement to improve the functioning of the urinary tract, and in cosmetics for its moisturizing and anti-inflammatory properties. Xylose is used for the production of furfural [40]. Rhamnose is used for the production of furanones, as a pharmaceutical precursor, and also for the production of savory flavors. Arabinose is a component containing various polysaccharides, gums, and hemicelluloses, while galacturonic acid is an acidifying agent in foods and a monomer of pectin molecules [41].

There are different methods that can be used to determine the composition of polysaccharides present in vegetables; among these methods, acid hydrolysis is one of the most commonly used for depolymerization of polysaccharides to break them down into monosaccharides [42,43]. This method requires a delicate balance between the acid concentration, type of acid, and temperature to avoid unwanted side products [44]. The resulting individual monosaccharides that make up the hydrocolloids of *O. ficus-indica* can be determined by several techniques, including chromatography, capillary electrophoresis, infrared spectroscopy (IR), light scattering detection, and nuclear magnetic resonance (NMR) spectroscopy. However, a disadvantage of these methods is that they require considerable financial investment and long processing times. One commonly used technique to identify the hydrolyzed polysaccharides is thin-layer chromatography (TLC); this technique has several advantages, such as the fact that it provides a reliable identification and sensitivity to less than 1 μg of monosaccharide, it is adaptable in the selection of the stationary and mobile phases, the sample preparation is simple, a considerable amount of the analyte can be evaluated, several samples can be analyzed simultaneously, chromatograms show a well-defined pattern for each compound, and solvent consumption is small. In addition, it allows the visualization of monosaccharides and qualitative chromatographic characteristics [43,44,45,46,47].

An accurate technique to determine the concentration of monosaccharides is the phenol–sulfuric acid method, which is a colorimetric method based on the reaction between a solution of hydrolyzed polysaccharides and a coloring reagent; this reagent is detectable in the visible range of the electromagnetic spectrum because it develops a yellow–orange color [48,49]. It is simple, quick, sensitive, reproducible, and specific for monosaccharides. The reagents are readily available and stable. All classes of monosaccharides, including oligo- and polysaccharides, can be determined using the phenol–sulfuric acid method [50,51].

In this research, we developed a fast and reliable methodology allowing us to identify and quantify the monosaccharides present in the hydrocolloids of the cactus *O. ficus-indica.* The identification of the monosaccharides was carried out by means of TLC, showing well-defined chromatograms that enabled an excellent comparison with the standards. The phenol–sulfuric acid method was used to quantify the release of monosaccharides through the hydrolysis process. With the proposed methodology, the hydrolysis time and the volume of the reagents were decreased considerably. The purpose of this work was to identify and quantify the hydrocolloid composition through simple and cost-effective techniques to provide us with a guide for techno-functional applications. The TLC and phenol–sulfuric acid methods are validated techniques allowing us to obtain results quickly and accurately [3,39,52,53,54,55,56].

We consider that the novelty of the methodology proposed here is that it is more efficient due to the reduced reaction times and less severe conditions. We achieved rapid and sensitive identification and quantification of the monosaccharides contained in the hydrolyzed samples.

## 2. Results and Discussion

The composition of the hydrocolloids obtained from the cactus is variable; it depends on several factors, such as the maturation time of the cladode, the place and time of harvest, and the extraction method used. Additionally, the monosaccharides integrated in the hydrocolloids are also variable; the biochemical processes of the cell wall and the hydrolysis process itself cause the interconversion of monosaccharides, which explains the presence of glucose, glucuronic acid, and fucose, among others [57].

**Figure 4:**
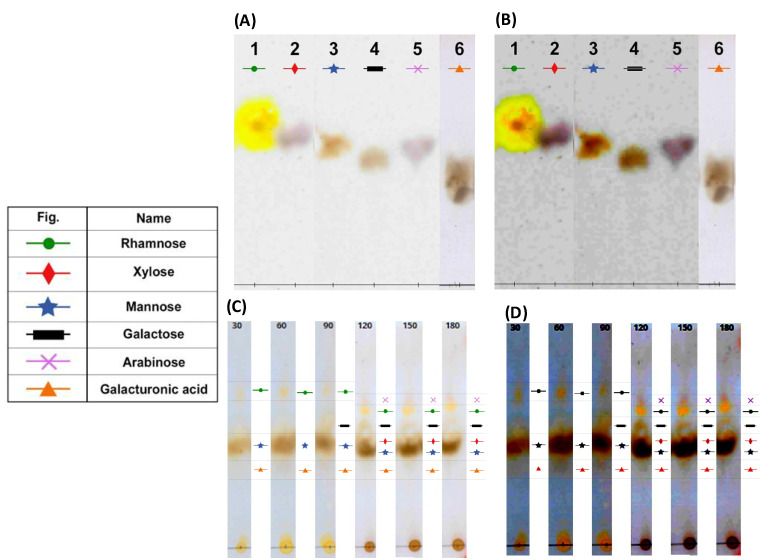
(**A**,**B**) The chromatograms from monosaccharide standards at (**A**) 5 min heat exposure and (**B**) 10 min heat exposure. (**C**,**D**) Cactus (*O. ficus-indica*) hydrocolloid samples of the hydrolysate at (**C**) 5 min heat exposure and (**D**) 10 min heat exposure.

The TLC conditions used here (the stationary and mobile phases and the developed chromatograms) allowed us to characterize the specific patterns of the monosaccharide standards (see Figure 4A,B). On the other hand, the acid hydrolysis conditions (i.e., type and concentration of the acid, temperature, and reaction conditions) were adequate to obtain distinctive chromatograms. The conditions used in both TLC and acid hydrolysis enabled the identification of the monosaccharides released in the depolymerization process (see Figure 4C,D). Table 1 shows the R_f_ of the standards used; the experiments were performed in triplicate, thus ensuring their correct use for comparison with the sample.

The importance of identifying the monosaccharides that make up the hydrocolloids lies in the fact that it enables elucidation of the properties of the polysaccharides present in the cactus (*O. ficus-indica)* and, thus, finding their functional application.

Figure 4C,D show the TLC of an acid-hydrolyzed sample of cactus (*O. ficus-indica)* hydrocolloid that was monitored every 30 min for a total period of 180 min. From 30 to 150 min, we observed the defined patterns of galacturonic acid (reported as the main monosaccharide), rhamnose (monosaccharides that form the main chain), and mannose.

At 90 min, the galactose pattern was gathered and rhamnose intensified, suggesting the formation of a rhamnose–galactose–xylose complex, which remained constant until 150 min. In contrast to carrying out the hydrolysis for 24 h using 3.9 M HCl, in this work, we reduced the time to 180 min using 2.5 N H_2_SO_4_. Both acids are known to prevent monosaccharide degradation.

**Figure 5:**
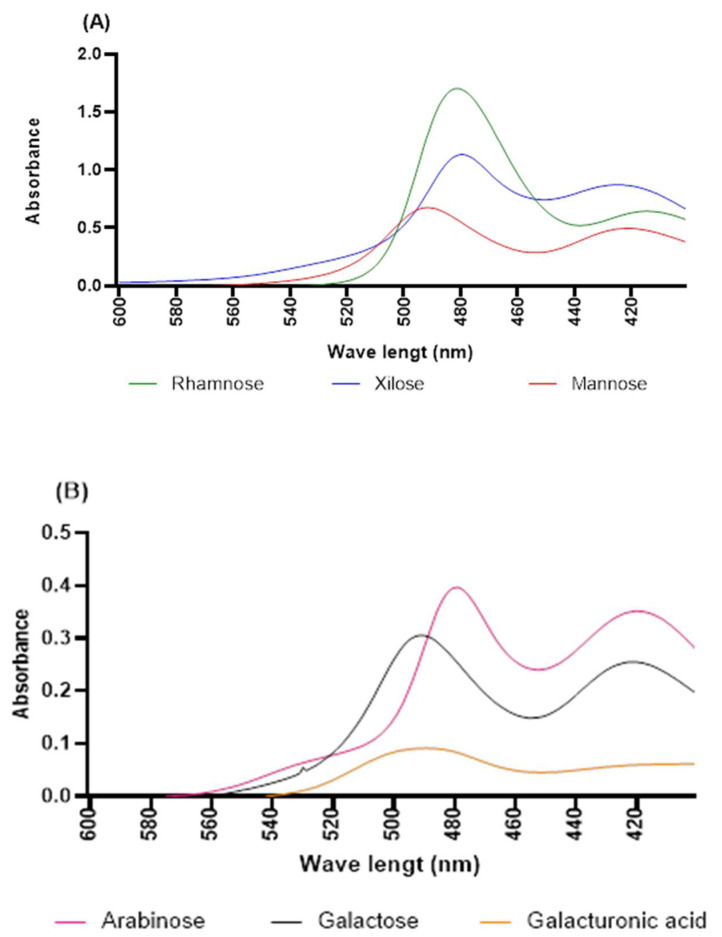
Spectra (600–420 nm) for the phenol–sulfuric acid reaction of monosaccharide standards: (**A**) rhamnose, xilose and mannose; (**B**) arabinose, galactose and galacturonic acid.

**Figure 6:**
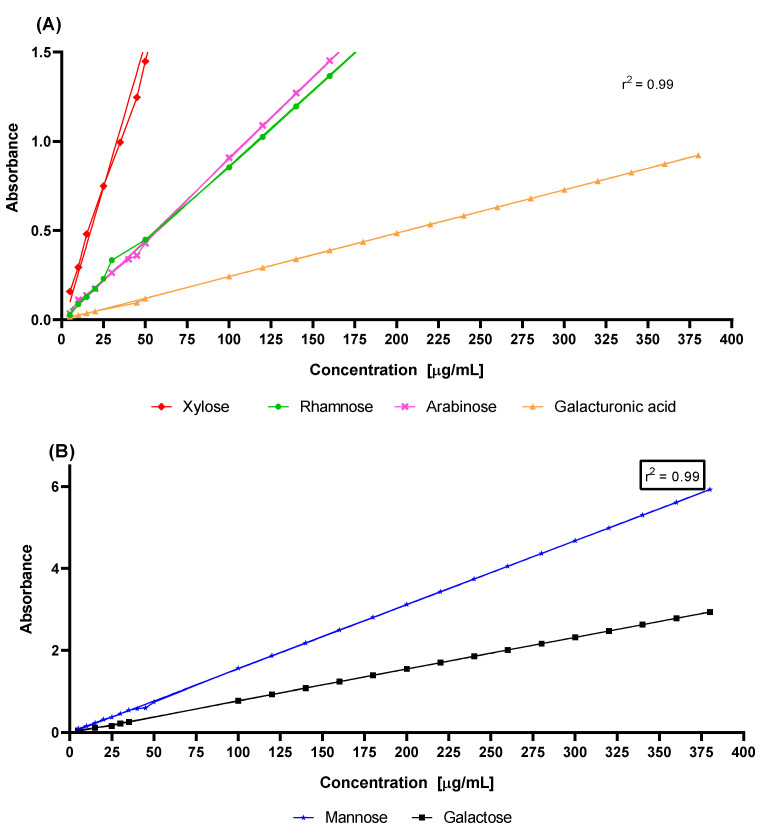
Calibration curves of monosaccharide standards in *O. ficus-indica* hydrocolloids: (**A**) xylose, rhamnose, arabinose and galacturonic acid; (**B**)mannose and galactose.

Figure 5 shows the light absorption in the entire visible range 600–420 nm of the electromagnetic spectrum for all of the monosaccharide standard solutions prepared using the phenol–sulfuric acid method but working with reduced reagent volumes. This figure depicts the fraction of the light absorbed (*y*-axis) as a function of wavelength (*x*-axis). Despite presenting a maximum at the same wavelength in nanometers, each standard of the monosaccharides showed a different spectral pattern, with the maximum between 480 and 495 nanometers [58]. In addition, the calibration curve of each monosaccharide that was evaluated is presented Figure 6. The results were analyzed by linear regression in order to obtain the coefficient of determination, r^2^ [59]. The correlation coefficients obtained in this work were 0.99, corroborating the linearity of the system and, therefore, allowing us to identify and calculate the concentrations of each of the hydrolyzed samples. 

**Figure 7:**
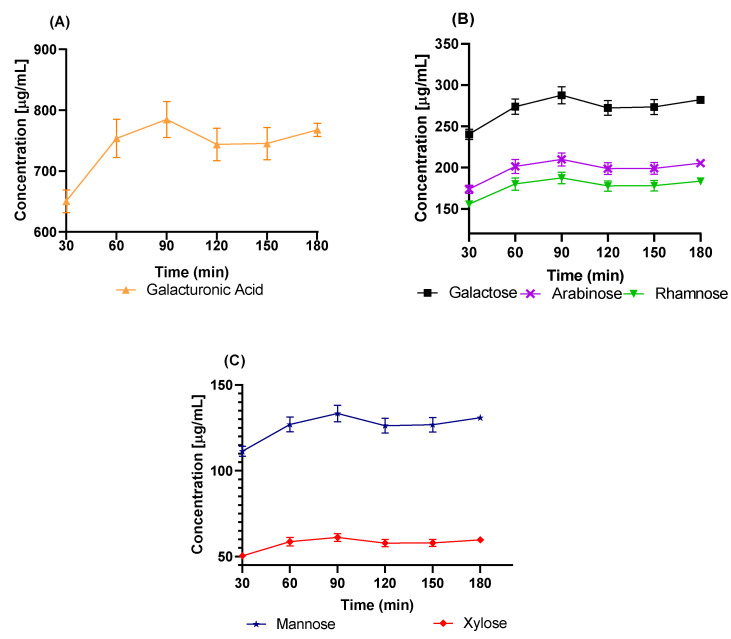
Concentration vs. time reaction of the monosaccharides present in *O. ficus-indica* hydrocolloids after acid hydrolysis: (**A**) galacturonic acid; (**B**) galactose, arabinose, and rhamnose; (**C**) mannose and xylose.

In other studies, Garna et al., [60] obtained a maximum concentration in the hydrolysis at different times for each monosaccharide. However, under the hydrolysis conditions used here, the maximum concentration of all of the evaluated monosaccharides was obtained at 90 min, as shown in Figure 7. Taking into consideration the experimental conditions of this work, we may attribute the high concentration of galacturonic acid observed in Figure 7 to its presence as the principal component in the main chains of pectin and mucilage (see Figure 2 and Figure 3, respectively). The second highest monosaccharide concentration was galactose, followed by arabinose; these two monosaccharides are linked to rhamnose, and are substituents in the ramifications of both pectin and mucilage. The lowest concentrations corresponded to xylose, which is the terminal of the ramifications present in the hydrocolloids.

In this work, the identification and quantification of the main monosaccharides reported in pectin and mucilage were carried out; however, we also detected small amounts of mannose (see Figure 7C), which is not reported as a constitutive monosaccharide of either pectin or mucilage. Table 2 shows the mean and SEAM values for every concentration versus hydrolysis time. We suggest that this monosaccharide comes from cell wall structures (Figure 1) present in the hydrocolloids due to the extraction process. We base this assumption on the fact that the mannose concentration was relatively low compared to those of the other monosaccharides [15,61,62,63]. These techniques provide us with a much faster analysis method, with lower consumption of chemical reagents, and without the need for expensive equipment. It is for all of the above reasons that we consider the results of this research to be of importance, since they result in a new methodology that is easy to use and cost-effective [64,65].

## 3. Materials and Methods

### 3.1. Materials

The dried and pulverized hydrocolloids of *O. ficus-indica* were provided by the ICAT-UNAM Process Engineering Laboratory. Concentrated sulfuric acid and phenol were obtained from J.T. Baker (Phillipsburg, NJ, USA). Monosaccharide standards L-rhamnose, D-xylose, D-mannose, L-arabinose D-galactose, and D-galacturonic acid, along with thin-layer chromatography silica gel matrix 60 A with medium pore diameter, n-butanol, and acetic acid, were all purchased from Sigma-Aldrich. The spectrophotometer was a Cary 5000 UV–VIS–NIR.

### 3.2. Methods

#### 3.2.1. Acid Hydrolysis of *O. ficus-indica* Hydrocolloids

The hydrocolloids were extracted from fresh cladodes (15 days) of *O. ficus-indica* collected in Milpa Alta, Mexico City, in 2018. The process is described by Reyes-Ocampo et al. [51]. The hydrolytic treatment procedure consisted of adding 0.5 g of the sample and 7.5 mL of 2.5 N H_2_SO_4_, and the reaction was maintained at 80 °C, taking aliquots of 100 µL every 30 min until completing a period of 180 min. The samples were cooled to 0 °C in an ice bath before analysis, to avoid degradation of the thin layer of silica.

#### 3.2.2. Thin-Layer Chromatography

For TLC, 0.01 g of each monosaccharide standard was dissolved in 1 mL of 2.5 N H_2_SO_4_. To follow the reaction, 3.0 μL of each hydrolyzed sample was deposited with a capillary on the thin layer of silica. The eluent solution for the standards and samples used was n-butanol, acetic acid, and distilled water at a 5:2.5:2.5 (*v/v*) ratio. The chromate plates were prepared in triplicate and developed with a solution of 2% sulfuric acid and water/EtOH at a 1:1 *v/v* ratio. To reveal the chromate plates, they were heated for 5 min at a temperature of 80 °C.

#### 3.2.3. Phenol–Sulfuric Acid Method

Once the hydrolysis was carried out, the quantification of the monosaccharide standards and samples was performed using the phenol–sulfuric acid method at reduced volumes [44,45,46,51]. The detailed procedure of the method employed in this study is as follows: A calibration curve was constructed as described by Dubois in 1956 [52] in triplicate, using the monosaccharides being assayed. Then, 500 µL of each sample was mixed with 300 µL of 5% phenol (*v/v*) in a test tube; 1.8 mL of concentrated sulfuric acid was added to the mixture, and then the test tube was cooled in an ice bath for 2 min and before being stored at room temperature for 15 min. The concentration of each sample was determined according to the standard curve. The absorption maxima were determined at 480 nm for pentoses (i.e., galacturonic acid, rhamnose, arabinose, and xylose) and 490 nm for hexoses (i.e., galactose and mannose). All regression analyses were performed using Prism (GraphPad Software, San Diego, CA, USA).

## 4. Conclusions

The acid hydrolysis conditions (2.5 N concentration of H_2_SO_4_, 80 °C temperature, and 180 min time) allowed the depolymerization of the cactus hydrocolloids (*O. ficus-indica*), shortening the reported hydrolysis times [58,59,60,61,62]. Likewise, under these conditions, the maximum concentration of all of the evaluated monosaccharides was obtained at 90 min, as shown in the time vs. concentration graph.

In this study, the TLC chromatograms showed a specific fingerprint of the monosaccharide standards, enabling a good comparison with the chromatograms of the hydrolyzed samples and, thus, achieving a qualitative analysis of the cactus (*O. ficus-indica*) hydrocolloids, while effectively identifying whether the monosaccharides of interest were present in the analyzed samples. Furthermore, the reduced-volume phenol–sulfuric acid method was accurate for the identification procedure, given that each monosaccharide exhibited a characteristic spectrum in the performed sweep (wavelength: 600 to 420 nm), and with the additional benefit of allowing smaller volumes of reagents, generating less waste.

For the evaluated samples, galacturonic acid was present at higher concentrations, since this monosaccharide comes from both the mucilage and the pectin of the prickly pear hydrocolloid (*O. ficus-indica*). The proportion of monosaccharides can vary according to the physiological needs of the plant, and this method makes it possible to know the concentration of monosaccharides at any time during the plant’s growth.

In conclusion, under the experimental conditions described above, a methodology was established consisting of (a) rapid depolymerization (optimizing the reaction time 72 h to 180 min), (b) simple identification by TLC, and (c) quantification via the phenol–sulfuric acid method. The described methodology enables a fast, reliable, and cost-effective procedure to depolymerize, identify, and quantify the monosaccharides that compose the hydrocolloids extracted from the cactus *O. ficus-indica*.

## Figures and Tables

**Table 1:** $R_{f}$ values for every monosaccharide standard (*n* = 3).

	Galacturonic Acid		Rhamnose		Galactose		Arabinose		Xylose		Mannose	
	Mean	SEAM	Mean	SEAM	Mean	SEAM	Mean	SEAM	Mean	SEAM	Mean	SEAM
$R_{f}$	0.4	0.006	0.47	0.015	0.39	0.044	0.40	0.009	0.43	0.013	0.4	0.009

**Table 2:** Mean and SEAM values for every monosaccharide.

	Galacturonic Acid		Rhamnose		Galactose		Arabinose		Xylose		Mannose	
Time	Mean	SEAM	Mean	SEAM	Mean	SEAM	Mean	SEAM	Mean	SEAM	Mean	SEAM
30	650.71	18.89	155.58	4.47	240.27	6.20	173.70	5.07	50.49	1.49	111.41	2.89
60	753.95	31.65	180.00	7.49	273.78	9.16	201.40	8.49	58.66	2.50	127.01	4.27
90	784.98	29.59	187.34	7.00	287.54	10.35	209.73	7.94	61.11	2.34	133.42	4.82
120	743.96	26.74	177.64	6.32	272.32	9.13	198.72	7.17	57.87	2.12	126.33	4.25
150	745.38	26.47	177.97	6.26	273.49	9.10	199.10	7.10	57.98	2.10	126.88	4.24
180	767.76	11.20	183.27	2.65	282.13	3.39	205.11	3.00	59.75	0.89	130.90	1.58

## Data Availability

Not applicable.

## References

[1] Pérez-Torrero E., Garcia-Tovar S.E., Luna-Rodriguez L.E., RodríguezGarcia M.E. (2017). Chemical Composition of Prickly Pads from *Opuntia ficus-indica* (L.) Miller Related to Maturity Stage and Environment. Int. J. Plant Biol. Res..

[2] Elkady W.M., Bishr M.M., Abdel-Aziz M.M., Salama O.M. (2020). Identification and Isolation of Anti-Pneumonia Bioactive Compounds from *Opuntia ficus-indica* Fruit Waste Peels. Food Funct..

[3] Contreras-Padilla M., Rodríguez-García M.E., Gutiérrez-Cortez E., Valderrama-Bravo M.D.C., Rojas-Molina J.I., Rivera-Muñoz E.M. (2016). Physicochemical and Rheological Characterization of Opuntia Ficus Mucilage at Three Different Maturity Stages of Cladode. Eur. Polym. J..

[4] Amin E.S., Awad O.M., El-Sayed M. (1970). The Mucilage of Opuntia ficus-indica Mill. Carbohydr. Res..

[5] McGarvie D., Parolis H. (1981). Methylation Analysis of the Mucilage of Opuntia ficus-indica. Carbohydr. Res..

[6] Tripodo M., Lanuzza F., Mondello F., Occhiuto F., Galati E. (2013). Enzymatic Extraction of Pectin from *Opuntia ficus-indica* Cladodes. VIII International Congress on Cactus Pear and Cochineal 1067.

[7] Bayar N., Bouallegue T., Achour M., Kriaa M., Bougatef A., Kammoun R. (2017). Ultrasonic Extraction of Pectin from *Opuntia Ficus Indica* Cladodes After Mucilage Removal: Optimization of Experimental Conditions and Evaluation of Chemical and Functional Properties. Food Chem..

[8] McGarvie D., Parolis H. (1979). The Mucilage of Opuntia ficus-indica. Carbohydr. Res..

[9] Haughn G.W., Western T.L. (2012). Arabidopsis Seed Coat Mucilage is a Specialized Cell Wall that Can be Used as a Model for Genetic Analysis of Plant Cell Wall Structure and Function. Front. Plant Sci..

[10] Espino-Díaz M., Ornelas-Paz J.D.J., Martínez-Téllez M., Santillán C., Barbosa-Cánovas G.V., Zamudio-Flores P.B., Olivas G.I. (2010). Development and Characterization of Edible Films Based on Mucilage of *Opuntia ficus-indica* (L.). J. Food Sci..

[11] Du Toit A., De Wit M., Hugo A. (2018). Cultivar and Harvest Month Influence the Nutrient Content of Opuntia spp. Cactus Pear Cladode Mucilage Extracts. Molecules.

[12] Phillips G.O., Williams P.A. (2009). Handbook of Hydrocolloids.

[13] Goycoolea F.M., Cárdenas A. (2003). Pectins from Opuntia spp.: A Short Review. J. Prof. Assoc. Cactus Dev..

[14] Nobel P.S., Cavelier J., Andrade J.L. (1992). Mucilage in Cacti: Its Apoplastic Capacitance, Associated Solutes, and Influence on Tissue 5. J. Exp. Bot..

[15] Buchanan B.B., Gruissem W., Jones R.L. (2015). Biochemistry and Molecular Biology of Plants.

[16] BeMiller J.N., Nielsen S.S. (2010). Carbohydrate Analysis. Food Analysis.

[17] Caffall K.H., Mohnen D. (2009). The Structure, Function, And Biosynthesis of Plant Cell Wall Pectic Polysaccharides. Carbohydr. Res..

[18] Tanczos I., Schwarzinger C., Schmidt H., Balla J. (2003). THM-GC/MS Analysis of Model Uronic Acids of Pectin and Hemicelluloses. J. Anal. Appl. Pyrolysis.

[19] Cárdenas Y., Ríos-Silva M., Huerta M., López M., Bricio-Barrios J., Ortiz-Mesina M., Urzúa Z., Saavedra-Molina A., Trujillo X. (2019). The Comparative Effect of Nopal and Mucilage in Metabolic Parameters in Rats with a High-Fructose Diet. J. Med. Food.

[20] Voragen A.G.J., Coenen G.-J., Verhoef R.P., Schols H.A. (2009). Pectin, a Versatile Polysaccharide Present in Plant Cell Walls. Struct. Chem..

[21] Burton R., Gidley M.J., Fincher G.B. (2010). Heterogeneity in the Chemistry, Structure and Function of Plant Cell Walls. Nat. Chem. Biol..

[22] Kumar S., Gupta S.K. (2012). Natural Polymers, Gums and Mucilages as Excipients in Drug Delivery. Polim. Med..

[23] Harholt J., Suttangkakul A., Vibe Scheller H. (2010). Biosynthesis of Pectin. Plant Physiol..

[24] Du Toit A. (2016). Selection, Extraction, Characterization and Application of Mucilage from Cactus Pear (Opuntia Ficus-Indica and Opuntia Robusta) Cladodes.

[25] Saag L.M.K., Sanderson G.R., Moyna P., Ramos G. (1975). Cactaceae Mucilage Composition. J. Sci. Food Agric..

[26] León-Martínez F., Méndez-Lagunas L., Rodríguez-Ramírez J. (2010). Spray Drying of Nopal Mucilage (Opuntia Ficus-Indica): Effects on Powder Properties and Characterization. Carbohydr. Polym..

[27] Bala R., Rana R., Madaan R. (2019). Natural Gums and Mucilage as Matrix Formers in Sustained Released Dosage Forms. Res. J. Pharm. Technol..

[28] Rodríguez-Garcia M.E., De Lira C., Hernández-Becerra E., Villegas M.D.L.A.C., Palacios A., Rojas-Molina I., Reynoso R., Quintero L.C., Del-Real A., Zepeda T.A. (2007). Physicochemical Characterization of Nopal Pads (*Opuntia ficus indica*) and Dry Vacuum Nopal Powders as a Function of the Maturation. Mater. Veg..

[29] Habtemariam S., Habtemariam S. (2019). Chapter 1—Type-2 Diabetes: Definition, Diagnosis and Significance. Medicinal Foods as Potential Therapies for Type-2 Diabetes and Associated Diseases.

[30] Guevara-Arauza J.C., Ornelas-Paz J.D.J., Pimentel D., Mendoza S.R., Guerra R.E.S., Maldonado L.M.T.P. (2012). Prebiotic Effect of Mucilage and Pectic-Derived Oligosaccharides from Nopal (*Opuntia ficus-indica*). Food Sci. Biotechnol..

[31] Sepúlveda E., Sáenz C., Aliaga E., Aceituno C. (2007). Extraction and Characterization of Mucilage in *Opuntia* spp.. J. Arid. Environ..

[32] Sáenz C., Sepúlveda E., Matsuhiro B. (2003). *Opuntia* spp Mucilage’s: A Functional Component with Industrial Perspectives. J. Arid Environ..

[33] de Araújo F.F., Farias D.D.P., Neri-Numa I.A., Pastore G.M. (2021). Underutilized Plants of the Cactaceae Family: Nutritional Aspects and Technological Applications. Food Chem..

[34] Soukoulis C., Gaiani C., Hoffmann L. (2018). Plant Seed Mucilage as Emerging Biopolymer in Food Industry Applications. Curr. Opin. Food Sci..

[35] Carmona J.C., Robert P., Vergara C., Sáenz C. (2020). Microparticles of Yellow-Orange Cactus Pear Pulp (*Opuntia ficus-indica*) with Cladode Mucilage and Maltodextrin as a Food Coloring in Yogurt. LWT.

[36] Rodríguez-González S., Martínez-Flores H.E., Chávez-Moreno C.K., Macías-Rodríguez L.I., Zavala-Mendoza E., Romo M.G.G., Chacón-García L. (2014). Extraction and Characterization of Mucilage from Wild Species of *O puntia*. J. Food Process Eng..

[37] Sandoval D.C.G., Sosa B.L., Martínez-Ávila G.C.G., Fuentes H.R., Abarca V.H.A., Rojas R. (2019). Formulation and Characterization of Edible Films Based on Organic Mucilage from Mexican *Opuntia ficus-indica*. Coatings.

[38] Gheribi R., Khwaldia K. (2019). Cactus Mucilage for Food Packaging Applications. Coatings.

[39] Guadarrama-Lezama A.Y., Castaño J., Velázquez G., Carrillo-Navas H., Alvarez-Ramírez J. (2018). Effect of Nopal Mucilage Addition on Physical, Barrier and Mechanical Properties of Citric Pectin-Based Films. J. Food Sci. Technol..

[40] Badui Dergal S. (2006). Química de Los Alimentos.

[41] Waldron K. (2009). Handbook of Waste Management and Co-Product Recovery in Food Processing.

[42] Shi H., Wan Y., Li O., Zhang X., Xie M., Nie S., Yin J. (2020). Two-Step Hydrolysis Method for Monosaccharide Composition Analysis of Natural Polysaccharides Rich in Uronic Acids. Food Hydrocoll..

[43] UÇAR G., Balaban M. (2003). Hydrolysis of Polysaccharides with 77% Sulfuric Acid for Quantitative Saccharification. Turk. J. Agric. For..

[44] Liu D., Tang W., Yin J.-Y., Nie S.-P., Xie M.-Y. (2021). Monosaccharide Composition Analysis of Polysaccharides from Natural Sources: Hydrolysis Condition and Detection Method Development. Food Hydrocoll..

[45] Yang X., Chen F., Huang G. (2020). Extraction and Analysis of Polysaccharide from Momordica Charantia. Ind. Crop. Prod..

[46] Zhang Z., Xiao Z., Linhardt R.J. (2009). Thin Layer Chromatography for the Separation and Analysis of Acidic Carbohydrates. J. Liq. Chromatogr. Relat. Technol..

[47] Jain A., Jain R., Jain S. (2020). Thin Layer Chromatography of Carbohydrates. Basic Techniques in Biochemistry, Microbiology and Molecular Biology.

[48] Chaplin M., Kennedy J.J.M.S. (1986). Monosaccharides.

[49] Zhang W.-H., Wu J., Weng L., Zhang H., Zhang J., Wu A. (2019). An Improved Phenol-Sulfuric Acid Method for the Determination of Carbohydrates in the Presence of Persulfate. Carbohydr. Polym..

[50] Masuko T., Minami A., Iwasaki N., Majima T., Nishimura S.-I., Lee Y.C. (2005). Carbohydrate Analysis by a Phenol–Sulfuric Acid Method in Microplate Format. Anal. Biochem..

[51] Niaz K., Khan F., Shah M.A. (2020). Analysis of Carbohydrates (Monosaccharides, Polysaccharides). Recent Advances in Natural Products Analysis.

[52] Wolfgong W.J. (2016). Chemical Analysis Techniques for Failure Analysis: Part 1, Common Instrumental Methods. Handbook of Materials Failure Analysis with Case Studies from the Aerospace and Automotive Industries.

[53] Bordiga M., Travaglia F., Meyrand M., German J.B., Lebrilla C.B., Coisson J.D., Arlorio M., Barile D. (2012). Identification and Characterization of Complex Bioactive Oligosaccharides in White and Red Wine by a Combination of Mass Spectrometry and Gas Chromatography. J. Agric. Food Chem..

[54] Xu R.-B., Yang X., Wang J., Zhao H.-T., Lu W.-H., Cui J., Cheng C.-L., Zou P., Huang W.-W., Wang P. (2012). Chemical Composition and Antioxidant Activities of Three Polysaccharide Fractions from Pinecones. Int. J. Mol. Sci..

[55] Li S., Cai W.-J., Wang W., Sun M.-X., Feng Y.-Q. (2020). Rapid Analysis of Monosaccharides in Sub-milligram Plant Samples Using Liquid Chromatography–Mass Spectrometry Assisted by Post-column Derivatization. J. Agric. Food Chem..

[56] De Lucia D., Manfredini S., Vertuani S., Bernardi T. (2016). New Insights into Sugar Characterization in Complex Plant Matrices by High-Performance Thin-Layer Chromatography. J. Liq. Chromatogr. Relat. Technol..

[57] Zhang Z., Khan N.M., Nunez K.M., Chess E.K., Szabo C.M. (2012). Complete Monosaccharide Analysis by High-Performance Anion-Exchange Chromatography with Pulsed Amperometric Detection. Anal. Chem..

[58] DuBois M., Gilles K.A., Hamilton J.K., Rebers P.A., Smith F. (1956). Colorimetric Method for Determination of Sugars and Related Substances. Anal. Chem..

[59] Wu L., Gao Y., Ren W.-C., Su Y., Li J., Du Y.-Q., Wang Q.-H., Kuang H.-X. (2021). Rapid Determination and Origin Identification of Total Polysaccharides Contents in Schisandra Chinensis by Near-Infrared Spectroscopy. Spectrochim. Acta Part A Mol. Biomol. Spectrosc..

[60] Garna H., Mabon N., Nott K., Wathelet B., Paquot M. (2006). Kinetic of the hydrolysis of pectin galacturonic acid chains and quantification by ionic chromatography. Food Chem..

[61] Ginestra G., Parker M.L., Bennett R.N., Robertson J., Mandalari G., Narbad A., Curto R.B.L., Bisignano G., Faulds C.B., Waldron K.W. (2009). Anatomical, Chemical, and Biochemical Characterization of Cladodes from Prickly Pear [*Opuntia ficus-indica* (L.) Mill.]. J. Agric. Food Chem..

[62] Salehi E., Emam-Djomeh Z., Askari G., Fathi M. (2018). *Opuntia ficus indica* Fruit Gum: Extraction, Characterization, Antioxidant Activity and Functional Properties. Carbohydr. Polym..

[63] Reyes-Ocampo I., Córdova-Aguilar M.S., Guzmán G., Blancas-Cabrera A., Ascanio G. (2018). Solvent-Free Mechanical Extraction of *Opuntia ficus-indica* mucilage. J. Food Process Eng..

[64] Texco A., De Pachuca U.P. (2018). Optimization of the Acid Hydrolysis of Cladodes of Opuntia Ficus-Indica by Response Surface Methodology. Rev. Mex. De Ing. Química.

[65] Santagata G., Cimmino A., Poggetto G.D., Zannini D., Masi M., Emendato A., Surico G., Evidente A. (2022). Polysaccharide Based Polymers Produced by Scabby Cankered Cactus Pear (*Opuntia ficus-indica* L.) Infected by *Neofusicoccum batangarum*: Composition, Structure, and Chemico-Physical Properties. Biomolecules.

